# Pulling the plug – halting cancer's theft of mitochondria

**DOI:** 10.18632/oncoscience.374

**Published:** 2017-11-02

**Authors:** Christopher R. Marlein, Lyubov Zaitseva, Stuart A. Rushworth

**Affiliations:** Norwich Medical School, The University of East Anglia, Norwich Research Park, NR4 7TJ, United Kingdom

**Keywords:** mitochondria, NOX2, tunnelling nanotubes, free fatty acids, acute myeloid leukemia

The mitochondria organelle has a crucial cellular role as a powerhouse generating energy in the currency of adenosine triphosphate (ATP). Mitochondria are known to be implicated in disease, where genetic defects in mitochondrial DNA (mtDNA) result in debilitating mitochondrial disease. Even though mutations in mtDNA are known to occur in cancer cells, the malignant cell still relies on mitochondrial based pathways to generate the vast quantity of ATP required to fuel the rapidly proliferating cell. This is increasingly relevant in acute myeloid leukemia (AML) where, contrary to the Warburg hypothesis, the malignant cell relies on oxidative phosphorylation rather than aerobic glycolysis. This revelation is due to these AML blasts having increased mitochondrial mass compared to their non- malignant counterparts [1] [2]. Recently these increased mitochondrial levels in the malignant cell have, by both ourselves [3] and the Griessinger lab [4], been attributed to mitochondrial transfer from bone marrow stromal cells (BMSC). Our research also takes this one step further by elucidating the mechanism and the stimulus behind the mitochondrial movement.

We showed that NOX2 activity on the AML blast stimulates mitochondrial transfer and also that the mitochondria move via AML-formed tunnelling nanotubes (TNTs) [3]. These two discoveries provide biological targets to block the movement of mitochondria via NOX2 inhibitors, such as diphenyleneiodonium chloride (DPI), or TNT inhibitors such as cytochalasin B. In addition, we have shown *in vitro* that DPI causes apoptosis in AML blasts cultured with BMSC, with no effect seen on normal CD34+ hematopoietic stem cells. We therefore believe targeting mitochondrial transfer provides a potential novel therapeutic angle for the treatment of AML, aimed at the malignancy specifically. However, the effect of NOX2 inhibitors have yet to be tested *in vivo* and is an important step in turning the therapeutic idea into reality. However, we do show that *in vivo* inhibition of NOX2 on AML blasts, through shRNA knockdown (KD), reduces tumour burden and disease progression. The survival of animals engrafted with NOX2 KD AML blasts have significantly increased survival times in addition to reduced mitochondrial levels, highlighting the beneficial effect of mitochondrial transfer to AML *in vivo*.

The bone marrow (BM) microenvironment of AML is known to be crucial in the survival of the malignancy, where ex vivo AML blasts undergo apoptosis which can be prevented by co-culture with BMSC [5]. Targeting the interactions between the bone marrow environment and the AML blast, may provide an alternative treatment option for AML. Moreover, the emergence of mitochondrial transfer in hematologic malignancies has highlighted a biological process which occurs to provide the energy production machinery to the cancer cell, cutting off this flow of mitochondria is envisaged to cause apoptosis in AML blasts.

The AML blasts are now capable of increased mitochondrial based energy production, as they have gained the extra metabolic machinery from their microenvironment. However, the malignant still needs to acquire the fuel to feed the imported mitochondria, which generates increased ATP levels. Recently our group has shown that adipocytes, from the bone marrow microenvironment, release free fatty acids (FFA) which are transferred to the AML blasts via the fatty acid binding protein 4 (FABP4) [6]. These FFA are transported into the mitochondria through carnitine palmitoyltransferase 1 (CPT1), which feeds β-oxidation to generate ATP. It is highly plausible that these FFA are the extra fuel that the BMSC derived mitochondria require to generate the high levels of ATP.

**Figure 1:**
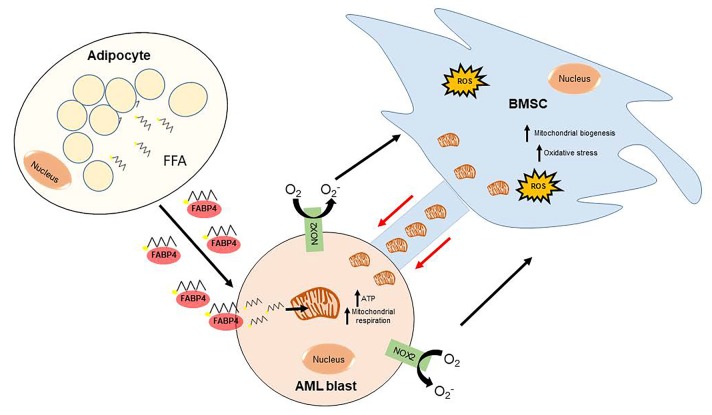
A schematic overview of how AML generates its metabolic ATP requirements The AML blast, through NOX2 activity, stimulates the mitochondrial transfer from the BMSC through tunnelling nanotubes (TNTs). This process can be inhibited with both NOX2 and TNT inhibitors. Free fatty acids (FFA) are released from adipocytes, in the bone marrow micro-environment, and are transferred to the AML blast via FABP4. These FFA enter the mitochondria, of BMSC origin, through CPT1 and generate ATP through β-oxidation. This provides the fuel and the metabolic machinery, which enables AML to generate the high ATP output it requires to rapidly proliferate.

AML blasts orchestrate the bone marrow microenvironment by getting BMSC to transfer their mitochondria and BM adipocytes to transfer their FFA. This allows the AML blast to cycle without control. Further studies to determine the impact of chemotherapy on FFA and mitochondrial transfer are now underway as it may provide an understanding of how AML blasts can survive under high doses of chemotherapy, within the protective microenvironment. The three cell system we describe is highlighted in schematic form in Figure 1.

We observe that AML requires both mitochondrial transfer and FFA transfer to generate the high ATP levels needed for its rapid proliferation. This highlights a potential dual therapeutic window, whereby the plug is pulled on both the fuel and machinery source leaving the blasts susceptible to chemotherapy regimens. Now the onus passes onto the pharmaceutical industry to develop compounds that target both mitochondrial transfer and FFA transfer. Mitochondrial transfer is however the most likely starting point as our recent publication in Blood, presents both NOX2 and TNTs, which could be targets for small molecule inhibitors.
